# Sir Benjamin Brodie

**Published:** 1858-09

**Authors:** 


SIR BENJAMIN BRODIE.—The Council of the Royal Society, London, have recommended Sir Benjamin Brodie to the Fellows as president of that most scientific body. As it is usual for those who are selected by the Council to be elected, Sir Benjamin will, in all probability, be placed at the head of a society of which he has always been a distinguished member. Not only is he the leading and most eminent surgeon of that great metropolis, but he also holds a high rank as a philosopher and a man of science, so that the Fellows of the society may feel proud of his election.

